# Unveiling the whole genomic features and potential probiotic characteristics of novel *Lactiplantibacillus plantarum* HMX2

**DOI:** 10.3389/fmicb.2024.1504625

**Published:** 2024-11-14

**Authors:** Tariq Aziz, Muhammad Naveed, Muhammad Aqib Shabbir, Abid Sarwar, Jasra Naseeb, Liqing Zhao, Zhennai Yang, Haiying Cui, Lin Lin, Thamer H. Albekairi

**Affiliations:** ^1^Department of Food Science and Technology, College of Chemistry and Environmental Engineering, Shenzhen University, Shenzhen, Guangdong, China; ^2^School of Biomedical Engineering, Shenzhen University, Shenzhen, Guangdong, China; ^3^Key Laboratory of Geriatric Nutrition and Health of Ministry of Education, Beijing Advanced Innovation Center for Food Nutrition and Human Health, Beijing Engineering and Technology Research Center of Food Additives, Beijing Technology and Business University, Beijing, China; ^4^School of Food and Biological Engineering, Jiangsu University, Zhenjiang, China; ^5^Department of Biotechnology, Faculty of Science and Technology, University of Central Punjab, Lahore, Pakistan; ^6^Department of Biotechnology, Faculty of Biological Sciences, Lahore University of Biological and Applied Sciences, Lahore, Pakistan; ^7^Department of Pharmacology and Toxicology, College of Pharmacy, King Saud University, Riyadh, Saudi Arabia

**Keywords:** comparative genomics, HMX2, coding sequences, food safety, *L. plantarum*

## Abstract

This study investigates the genomic features and probiotic potential of *Lactiplantibacillus plantarum* HMX2, isolated from Chinese Sauerkraut, using whole-genome sequencing (WGS) and bioinformatics for the first time. This study also aims to find genetic diversity, antibiotic resistance genes, and functional capabilities to help us better understand its food safety applications and potential as a probiotic. *L. plantarum* HMX2 was cultured, and DNA was extracted for WGS. Genomic analysis comprised average nucleotide identity (ANI) prediction, genome annotation, pangenome, and synteny analysis. Bioinformatics techniques were used to identify CoDing Sequences (CDSs), transfer RNA (tRNA) and ribosomal RNA (rRNA) genes, and antibiotic resistance genes, as well as to conduct phylogenetic analysis to establish genetic diversity and evolution. The study found a significant genetic similarity (99.17% ANI) between *L. plantarum* HMX2 and the reference strain. Genome annotation revealed 3,242 coding sequences, 65 tRNA genes, and 16 rRNA genes. Significant genetic variety was found, including 25 antibiotic resistance genes. A phylogenetic study placed *L. plantarum* HMX2 among closely related bacteria, emphasizing its potential for probiotic and food safety applications. The genomic investigation of *L. plantarum* showed essential genes, including plnJK and plnEF, which contribute to antibacterial action against foodborne pathogens. Furthermore, genes such as MurA, Alr, and MprF improve food safety and probiotic potential by promoting bacterial survival under stress conditions in food and the gastrointestinal tract. This study introduces the new genomic features of *L. plantarum* HMX2 about specific genetics and its possibility of relevant uses in food security and technologies. These findings of specific genes involved in antimicrobial activity provide fresh possibilities for exploiting this strain in forming probiotic preparations and food preservation methods. The future research should focus on the experimental validation of antibiotic resistance genes, comparative genomics to investigate functional diversity, and the development of novel antimicrobial therapies that take advantage of *L. plantarum*'s capabilities.

## 1 Introduction

The development of molecular techniques such as whole-genome sequencing (WGS) has transformed bacterial strain typing, significantly impacting epidemiological monitoring and outbreak analysis. Genomics and whole-genome sequencing (WGS) can significantly improve our understanding of infectious diseases and clinical microbiology (Avershina et al., 2023). The development of benchtop WGS analyzers has made genomics more accessible to clinicaland public health experts in microbiology. Despite the restrictions of resources and infrastructure, WGS is especially beneficial in public health laboratories, reference labs, and hospital infection control labs (Aziz et al., 2023a; Beltrán-Velasco et al., 2024). *Lactiplantibacillus plantarum* (previously known as *L. plantarum*) is a Gram-positive bacterium that lives in a variety of environments, including fermented dairy products, sourdough, fruits, vegetables, cereals, meat, fish, and the mammalian gastrointestinal tract. It is commonly employed as a starting culture in a variety of fermented foods, improving its flavor, texture, and sensory qualities (Beltrán-Velasco et al., 2024). The most frequently investigated strain is *L. plantarum* 299v, which has appeared in over 170 scholarly papers, including more than 60 human clinical investigations. *L. plantarum*'s genome is 3.3 Mb, larger than the other LAB species' typical 2–2.7-Mb genome, indicating high genetic diversity. Studies on six strains of *L. plantarum* revealed significant differences in prophages, transposases, IS elements, and plantaricin biosynthesis genes, along with notable variations in capsular and extracellular polysaccharide biosynthesis genes (Umanets et al., 2023).

This study aims to investigate the *L. plantarum* genome, as well as the identification of bacterial strain, sequence similarity comparison, gene annotation, and, finally, to perform a comparative analysis. The specific tools employed to analyze *L. plantarum* strains include ANI calculator, MetaGeneMark, Rapid Annotations using Subsystems Technology (RAST) toolkit, integrated prokaryotic genome analysis (IPGA) platform, OrthoVenn3 server, the Pathosystems Resource Integration Center (PATRIC), and the Search Tool for the Retrieval of Interacting Genes/Proteins (STRING) database. This study not only includes the comparison of nucleotide sequences with the other strain's orthologous genes but also includes the identification of the specialty genes and protein–protein interactions (PPIs), so this work provides a comprehensive understanding of the genomic complexness and evolutionary relationships within this bacterial species. All the information from this research proves useful in further studies related to microbial genetics, biotechnology, and pathogens functioning and diversification (Syaputri et al., 2023). Additionally, the project seeks to investigate genes associated with defense and survival to offer a thorough understanding of the strain's genetic composition and adaptive strategies. This research will enhance our knowledge of *L. plantarum*'s functional capabilities and potential applications in various industries. Investigating its genetic diversity may reveal insights that could derive novel biotechnological innovations (Rajput et al., 2023).

The study enhances understanding of *L. plantarum*'s genetic diversity and potential applications (Liu et al., 2023). It intended to discover and characterize *L. plantarum*, emphasizing its high genomic similarity, significant genetic diversity, and presence of critical defense and survival genes. The comprehensive genomic research broadens our understanding of *L. plantarum's* functional capabilities and prospective uses in food safety. The future studies will focus on the strain's abilities for adaptation across multiple environments, its potential in probiotic formulations, and its appropriateness for generating novel antimicrobial drugs.

## 2 Materials and methods

### 2.1 Bacterial strain identification and culturing

*L. plantarum* can be of great commercial importance owing to its possible uses in food and pharmaceutical fields. It has reported positive effects on gut health and immune systems and is used in biopreservation due to its capability for the natural preservation of fermentation products and for creating healthy functional foods (Hu et al., 2023). The target strain, *L. plantarum*, HMX2 was injected in deMan Rogosa Sharp (MRS) medium and grown for 20 h. The bacterial DNA extraction was carried out according to the “Bacterial Genomic DNA Extraction Kit (Beijing Trangen)” instructions by Beijing Tiangen. In a microcentrifuge tube, 0.5–4.0 ml of cells (maximum 2 × 10^9^ cells) were harvested by centrifugation for 1 min at maximum speed, with the supernatant discarded as much as possible. The pellet was resuspended in 100 μl of erythrocyte lysis (EL) Buffer and stirred thoroughly using a tip. The mixture was then incubated at 37°C for 40 min, with certain bacteria requiring an additional hour or more (Sadanov et al., 2023).

### 2.2 Whole-genome sequencing

The genomic DNA of *L. plantarum* HMX2 was sequenced by WGS with the HiSeq system of Illumina, and its coverage reached 12.0 times. The genomic DNA sequence assembly was done using the ABySS v.12.1 making it a circular genome with a size of 3,322,298 base pairs. Identification of genes and non-coding RNAs was done using the National Center for Biotechnology Information Prokaryotic Genome Annotation Pipeline (NCBI PGAP) for prokaryotic genomes; the resulting annotation included 3,172 genes, such as CoDing Sequences (CDSs), ribosomal RNAs (rRNAs), transfer RNAs (tRNAs), and ncRNAs. As a result of this high-quality assembly and annotation, aspects of the genomic organization of the strain are described comprehensively. The genome sequence was then submitted to NCBI under the allocated Accession No. GCF_025144505.1.

### 2.3 Average nucleotide identity prediction

To measure the nucleotide level genomic similarity of the genomes, the average nucleotide identity (ANI) prediction was performed by ANI Calculator available at EZCloud Server (https://www.ezbiocloud.net/tools/ani) (Yoon et al., 2017). The genome of *L. plantarum* Z.6-1 was utilized as a reference for ANI calculation. The reference genome was retrieved from National Centre for Biotechnology Information (NCBI) by specifically allocated accession number GCA_023973045.1. Both genomes were uploaded in FASTA QC format at ANI calculator and the analysis was performed. The results were analyzed for further interpretations (de Albuquerque and Haag, 2023).

### 2.4 Genome annotation and pan-genome analysis

The annotation of the genome was performed by MetaGenMark (https://genemark.bme.gatech.edu/meta_gmhmmp.cgi) (Jahanshahi et al., 2023). For this integrated genome analysis, the genomic information of *L. plantarum* HMX2 was retrieved from the Bacterial and Viral Bioinformatics Resource Center (BV-BRC) database (Olson et al., 2023). In this case, the genome of *L. plantarum* HMX2 was first identified, and then the FASTA files were downloaded. These quality control checks were done using the tools available in the BV-BRC (https://www.bv-brc.org) to see that all the sequences were accurate and complete. In the present investigations, genome annotations were performed employing the integrated RAST toolkit, which delineates comprehensive information regarding the gene functions and metabolic pathways. Numerous tools available at BV-BRC were utilized to compare multiple strains and detect some conserved and strain-specific genomic aspects. To arrive at valid conclusions about the kind of genome and the phylogenetic relatedness of the observed organisms, the results were assimilated and expounded in light of prior biological insights (Horsfield et al., 2023).

Pangenome and gene synteny were compared with the IPGA platform v1 (Liu et al., 2022). The quality of the genome assemblies was first assessed before gene clustering to determine the orthologous group. IPGA v1.09 integrated prokaryotes genome and pan-genome analysis service revealed differences in the gene content and its functional categorization at the scale of pangenome. To investigate the degree of gene order conservation, which sheds light on the evolutionary relationship, synteny analysis was done. The visualization of the genomic and synteny features was performed by IPGA visualization tools (Wang et al., 2023).

### 2.5 Orthologous analysis

The five *L. plantarum* strains (BDGP2, DF, HMX2, SRCM100442, and UNQLp11) were compared with Homology comparison of Orthologous Groups of proteins using the OrthoVenn3 server (https://orthovenn3.bioinfotoolkits.net) (Sun et al., 2023). First, the gene call for each strain was extracted from the respective genome assembly to obtain protein sequences. These sequences were then uploaded to the OrthoVenn3 server. The parameters for the analysis were kept to their default settings, which for identification of orthologs are an E-value of 1e-2 and an inflation value of 1.5 per clustering. The parameter for the k-means clustering was set to 5 as per the input of the clustering algorithm. In OrthoVenn3, all the protein sequences are compared to one another directly employing DIAMOND that subsequently groups the homologous clusters (Wang et al., 2015).

The Venn diagram was obtained to illustrate the degree of conservation and differences in the orthologous clusters among the five strains. The figure illustrates the number of clusters as well as the particular count of proteins belonging to every strain of the bacteria, and the overlapping of the clusters. This encapsulation, which depicts the genome, increasingly highlighted the fundamental elements of the virus along with the strain particular features. From here, the clusters were subdivided to deduce functional concerns of the conserved genes and those unique to *L. plantarum*, adding a clearer picture of functional conservation and diversification in *L. plantarum*. A comprehensive comparative genomics analysis of *L. plantarum* strains was conducted using OrthoVenn3, incorporating both a broad and narrow selection of strains.

### 2.6 Prediction of specialty genes

The sequencing data analysis for identifying specialty genes in *L. plantarum* HMX2 was performed on PATRIC () (Snyder et al., 2007). First, the WGS of *L. plantarum* HMX2 was uploaded to Pathosystems Resource Integration Center (PATRIC), and the sequences were automatically annotated and analyzed in Comprehensive Genome Analysis. The relevant bioinformatics tools available in the platform's databases were used to analyze and identify the new genes belonging to the categories of interest, namely, antibiotic resistance, transporters, or functional capabilities (Vidulin et al., 2016).

### 2.7 Protein interaction network

To map the selected proteins from *L. plantarum* HMX2 on the PPI network, the STRING database version 11. 5 (https://string-db.org) was used to determine the possible interaction (von Mering et al., 2003). The selected proteins for the PPI study were glpF6, fusA2, glpF1, rho, orf2, yidC1, and tuf. Each protein was submitted to enter STRING, which combines manually assembled and computationally predicted PPIs from various databases, interactions derived from experimental evidence, gene organizations, gene fusion, phylogenetic tree, co-occurrence, text references, gene expression profiles, and sequence similarity (Szklarczyk et al., 2015). To obtain the PPI data analysis and gene occurrence, the protein identifiers were entered into the STRING interface with an interaction score set to a medium confidence level of 0.400 to capture both high and moderate PPIs. The obtained network was then enlarged to emphasize the mutual connectivity of the query proteins and their first coordination sphere. The nodes are the individual proteins, whereas the edges are the anticipated connectivity between the proteins. The interactions were classified according to the evidence types such as, gene expression, experimental taxonomy, direct database, annotations, infrastructural databases, and integrated computational prediction. The visualization differentiated between proteins that had known or predicted structures and those with undetermined structures (filled nodes and empty nodes, respectively) (Al-Aamri et al., 2019; Aziz et al., 2023b).

## 3 Results

### 3.1 Average nucleotide identity prediction

The ANI Prediction was conducted using the ANI Calculator available at the EZ Cloud Server (https://www.ezbiocloud.net/tools/ani) to measure the nucleotide-level genomic similarity between two genomes. The genome of *L. plantarum* Z.6-1, retrieved from NCBI with accession number GCA_023973045.1, was used as the reference. Both genomes were uploaded in FASTA QC format for analysis. Genome sequence A (*L. plantarum* HMX2) had a total length of 3,322,298 bp with a GC content of 44.51%, while Genome sequence B (*L. plantarum* Z.6-1) had a total length of 3,333,079 bp with a GC content of 44.42%. The OrthoANIu analysis revealed an OrthoANIu value of 99.17%, with Genome A and B lengths of 3,322,140 and 3,332,340 bp, respectively. The average aligned length was 2,204,278 bp, with coverage of 66.35% for Genome A and 66.15% for Genome B. These results indicate a high level of genomic similarity between the analyzed genomes. The detailed results of the ANI calculation are given in Table 1.

**Table 1:** The average nucleotide identity (ANI) of *L. plantarum* HMX2 genome.

**Metric**	**Value**
OrthoANIu value (%)	99.17
Genome A length (bp)	3,322,140
Genome B length (bp)	3,332,340
Average aligned length (bp)	2,204,278
Genome A coverage (%)	66.35
Genome B coverage (%)	66.15

The findings from the complete genome analysis offered by the BV-BRC tool offered overall information regarding the genome's general features and alignment statistics; these results were obtained by including the genomes provided above. The analysis comprised 11 genomes; the number of genes in all the genomes varied between 1,126 and 2,659, single-copy genes varied between 532 and 683 with five filtered single copy-genes in all the genomes.

### 3.2 Genome annotation and genome assembly

PATRIC was used to analyze all the facets of *L. plantarum* HMX2 complete genome. The submitted assembled genome provided a single contig with a combination of base pairs 3,322,298 and ~44% G + C 51%. No plasmids or chromosomes were detected. The genome annotations were done using the RAST toolkit known as RASTtk. Table 2 represents the assembly details for the genome of *L. plantarum* HMX2.

**Table 2:** The assembly details of the *Lactiplantibacillus plantarum* HMX2 genome obtained from RASTtk.

**Assembly details**	
Contigs	1
GC content	44.51
Plasmids	0
Contig L50	1
Genome length	3,322,298 bp
Contig N50	3,322,298
Chromosomes	0

### 3.3 Genome annotation

Annotating the genome exposed 3,242 CDS, 65 tRNA genes, and 16 rRNA genes. Out of the 2,242 protein-coding genes, only 1,424 genes were hypothetical, and 1,818 genes had some sort of function annotation; 683 Enzyme Commission (EC) numbers, 577 Gene Ontology (GO) terms, and 484 genes had Kyoto Encyclopedia of Genes and Genomes (KEGG) Orthology mapping. No PLFams were found, but 3,143 of the proteins fell into cross-genus protein families or PGFams. Table 3 represents the annotated genome features of *L. plantarum* HMX2 genome, and the Table 4 represents the protein features of the genome.

**Table 3:** Annotated genome features of *Lactiplantibacillus plantarum* HMX2 genome.

**Annotated genome features**	
CDS	3,242
Repeat regions	71
tRNA	65
rRNA	16
Partial CDS	0
Miscellaneous RNA	0

CDS, CoDing Sequences; Trna, transfer RNA; rRNA, ribosomal RNA.

**Table 4:** Protein features of *Lactiplantibacillus plantarum* HMX2 genome.

**Protein features**	
Hypothetical proteins	1,424
Proteins with functional assignments	1,818
Proteins with EC number assignments	683
Proteins with GO assignments	577
Proteins with pathway assignments	484
Proteins with PATRIC genus-specific family (PLfam) assignments	0
Proteins with PATRIC cross-genus family (PGfam) assignments	3,143

**Figure 1:**
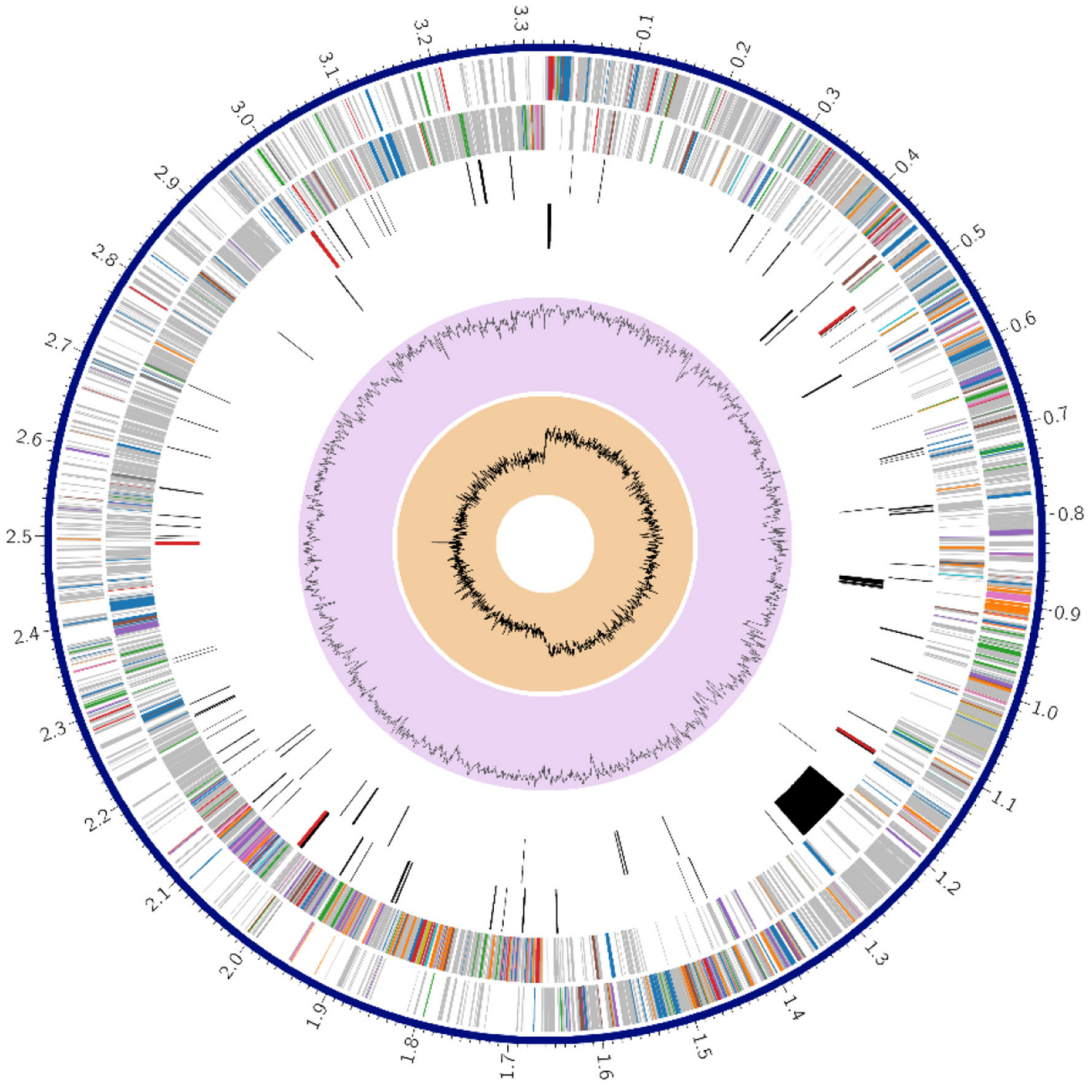
Circular genome annotation of *Lactiplantibacillus plantarum* HMX2 genome.

Figure 1 shows a circle chart showing the various genome annotations, such as CDS on the forward and reverse strands in red; RNA genes in violet; ARGs shown in green; VF shown in yellow. The guanine-cytosine (GC) content map is shown in blue and the GC skew map is shown in magenta. Metabolism comprised 63 genes; protein processing 37; stress response, defense, and virulence 22; DNA processing 18, energy 15 and RNA processing 12; cellular processes 9; membrane transport 6; cell envelope 4; regulation and cell signaling 3; and the remaining gene fell under miscellaneous category.

### 3.4 Subsystem analysis

**Figure 2:**
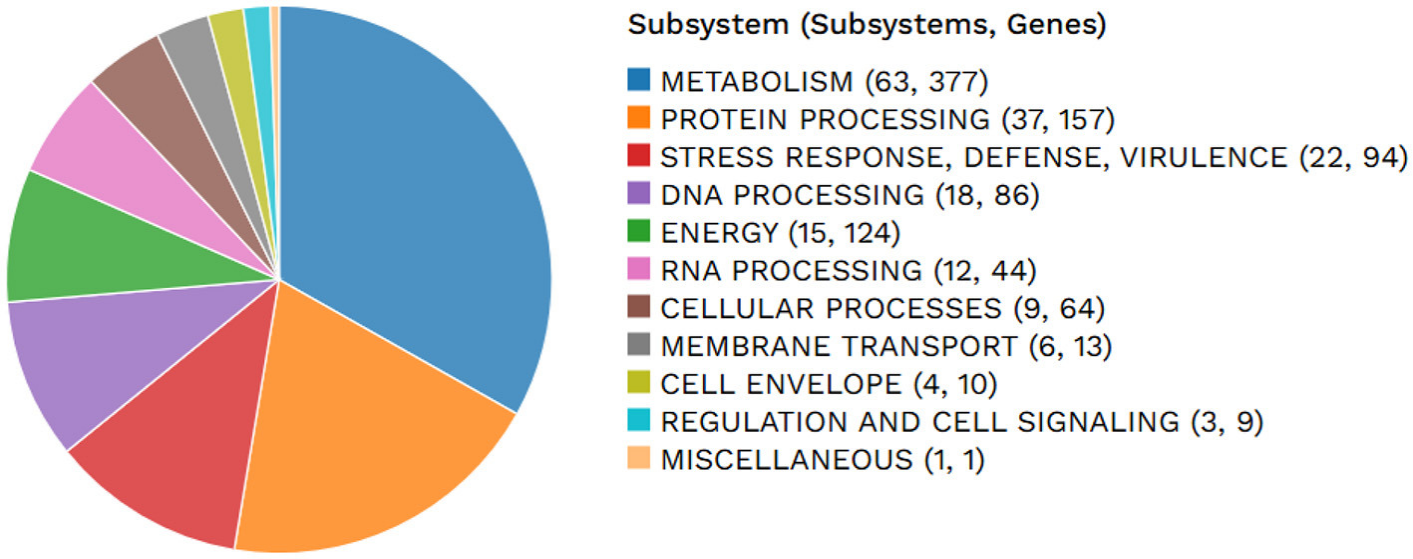
An overview of the subsystems in *Lactiplantibacillus plantarum* HMX2 genome.

In this systematic analysis of specialty genes, there were 25 genes that were detected to have a relation with antibiotic resistance, one drug target, and 14 transporters were detected (Table 5; Figure 2). Some of the categories of antimicrobial resistance (AMR) genes were the target modification genes that encode efflux pumps and the carbapenemase encoding genes. Therefore, other genes associated with crucial processes, such as metabolism, membrane transport, and protein processing, were also detected.

**Table 5:** The presence of specialty genes in *Lactiplantibacillus plantarum* HMX2 genome.

	**Source**	**Genes**
**Specialty genes**		
Antibiotic resistance	PATRIC	25
Drug target	DrugBank	1
Transporter	TCDB	14
**AMR mechanism**	**Genes**	
**Antimicrobial resistance genes**		
Antibiotic target in susceptible species	Alr, Ddl, EF-G, EF-Tu, folA, Dfr, folP, gyrA, gyrB, inhA, fabI, Iso-tRNA, kasA, MurA, rho, rpoB, rpoC, S10p, S12p	
Antibiotic target modifying enzyme	RlmA(II)	
Antibiotic target replacement protein	FabK	
Gene conferring resistance via absence	gidB	
Protein-altering cell wall charge conferring antibiotic resistance	GdpD, MprF, PgsA	

AMR, antimicrobial resistance; PATRIC, Pathosystems Resource Integration Center; TCDB, Transporter Classification Database.

### 3.5 Identification of antibiotic resistance genes

AMR gene identification in Genome Annotation Service in PATRIC was performed using k-mer based approach and functional descriptions and classifications were made. In this technique, AMR gene variants in PATRIC's library were classified into mechanisms of resistance, drug classes, and associated antibiotics (Supplementary Table S1). For instance, genes Alr, Ddl, and folA—direct the antibiotic susceptibility factors, and the genes gyrA and gyrB alter the antibiotic targets. Moreover, factors that modify charges, such as GdpD gene or MprF gene antibiotic resistance, change the charges of the cell wall proteins. At the same time, it should be mentioned that the presence of full-length genes related to AMR does not always mean the presence of AMR phenotype and, thus, the prerequisite for correlating the specific AMR mechanisms and the impact of SNP mutations.

### 3.6 Phylogenetic analysis

The phylogenetic analysis was designed to advance knowledge of the evolutionary history and genetic diversity of *L. plantarum* strains regarding their ecological niches and linkage to AMR. This study provides a foundation for the future directions in microbial genomics and its potential uses. Mash/MinHash and RaxML are the tools that help to understand the evolutionary history or phylogenetic relationships of the genome under study. To compare these strains and infer the location in the phylogenetic tree, additional tools were applied to this study to outline the closest reference and the representative genomes, and their protein families were predicted as PGFams. For the protein sequences derived from those families, MUltiple Sequence Comparison by Log-Expectation (MUSCLE) was applied for the sequences' alignment. In contrast, the nucleotide sequences' alignment was incorporated into a fused data matrix. Rate of Divergence (ROD) was then used for stable tree construction. Several types of such comprehensive phylogenetic analyses significantly expand our knowledge of the genetic variance and the processes of evolution within microbial taxa serving as a fundamental basis for further studies in the field of AMR and microbial economics.

### 3.7 Pangenome and genome synteny analysis

**Figure 3:**
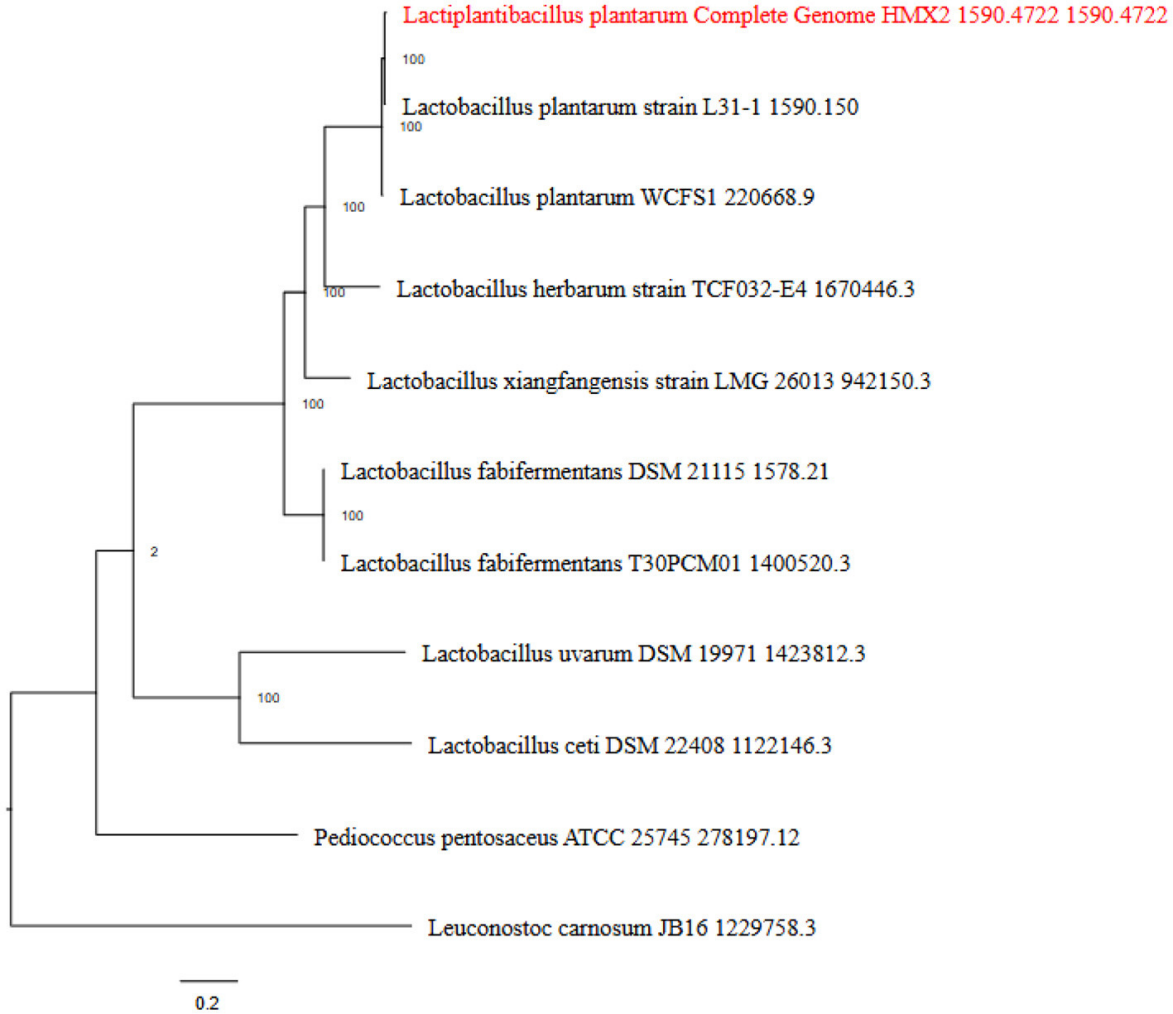
The phylogenetic tree indicating the ancestral relationship among various strains of *L. plantarum* HMX2 genome.

**Figure 4:**
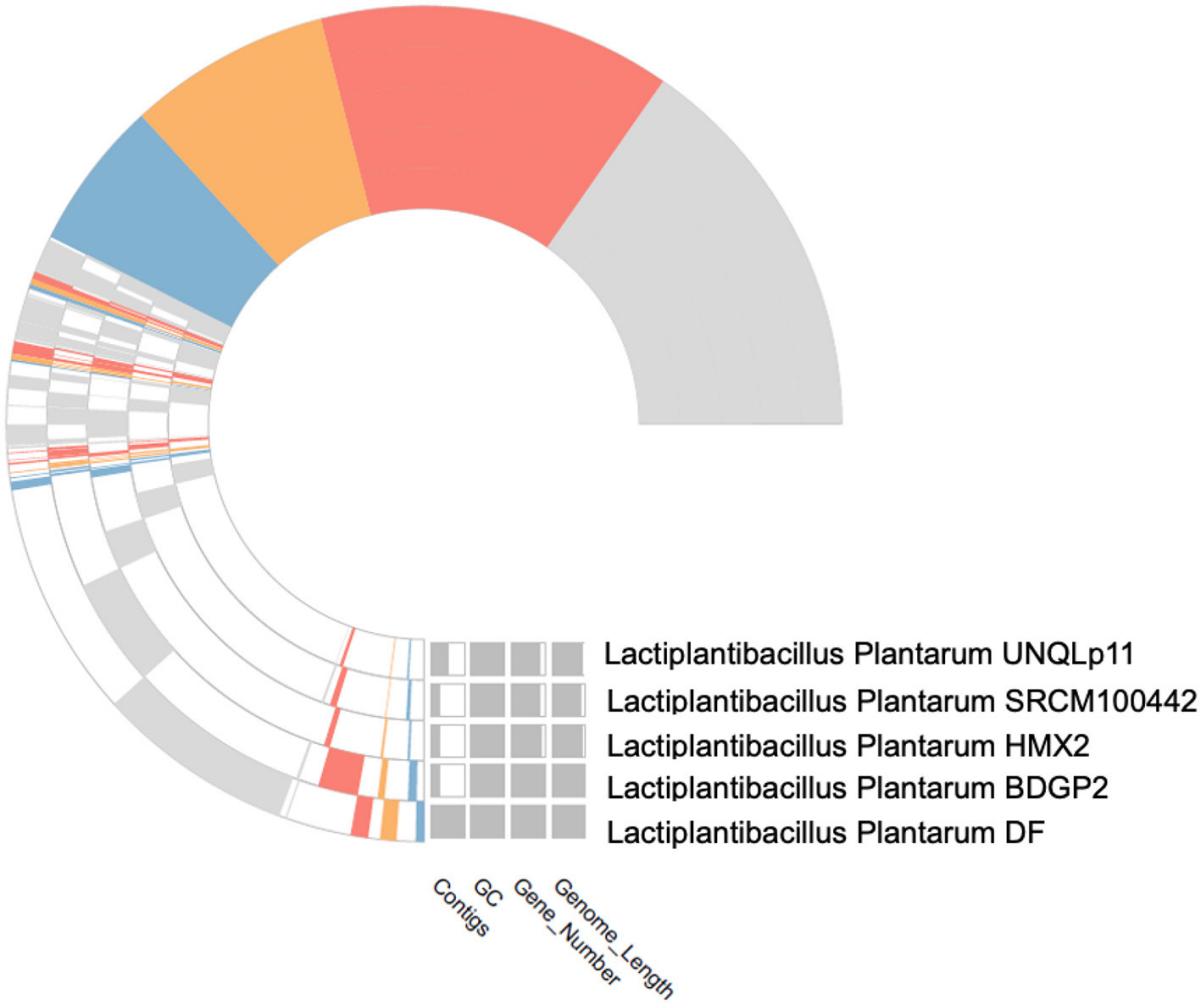
The cluster core analysis results obtained by PanGenome analysis.

Based on the IPGA v1 platform, several pangenomic analyses were performed. Several highly informative details about the pangenomic content of different genomes were identified and analyzed for the particular set of genomes. First, the correctness of the assemblies was evaluated to exclude low quality assemblies and obtain accurate data for further analyses. After that, gene clustering was used to identify ortholog groups, and this analysis showed the differences in gene content and its classification according to function within the pangenome. Hence, the IPGA offered better visualization aids regarding such genomic differences and inclusively improved the general genetic understanding of the specified genomes. The Figure 3 represents the Cluster Orthologous Groups (COGs) results obtained by the pangenome analysis. The cluster core analysis results are shown in Figure 4.

**Figure 5:**
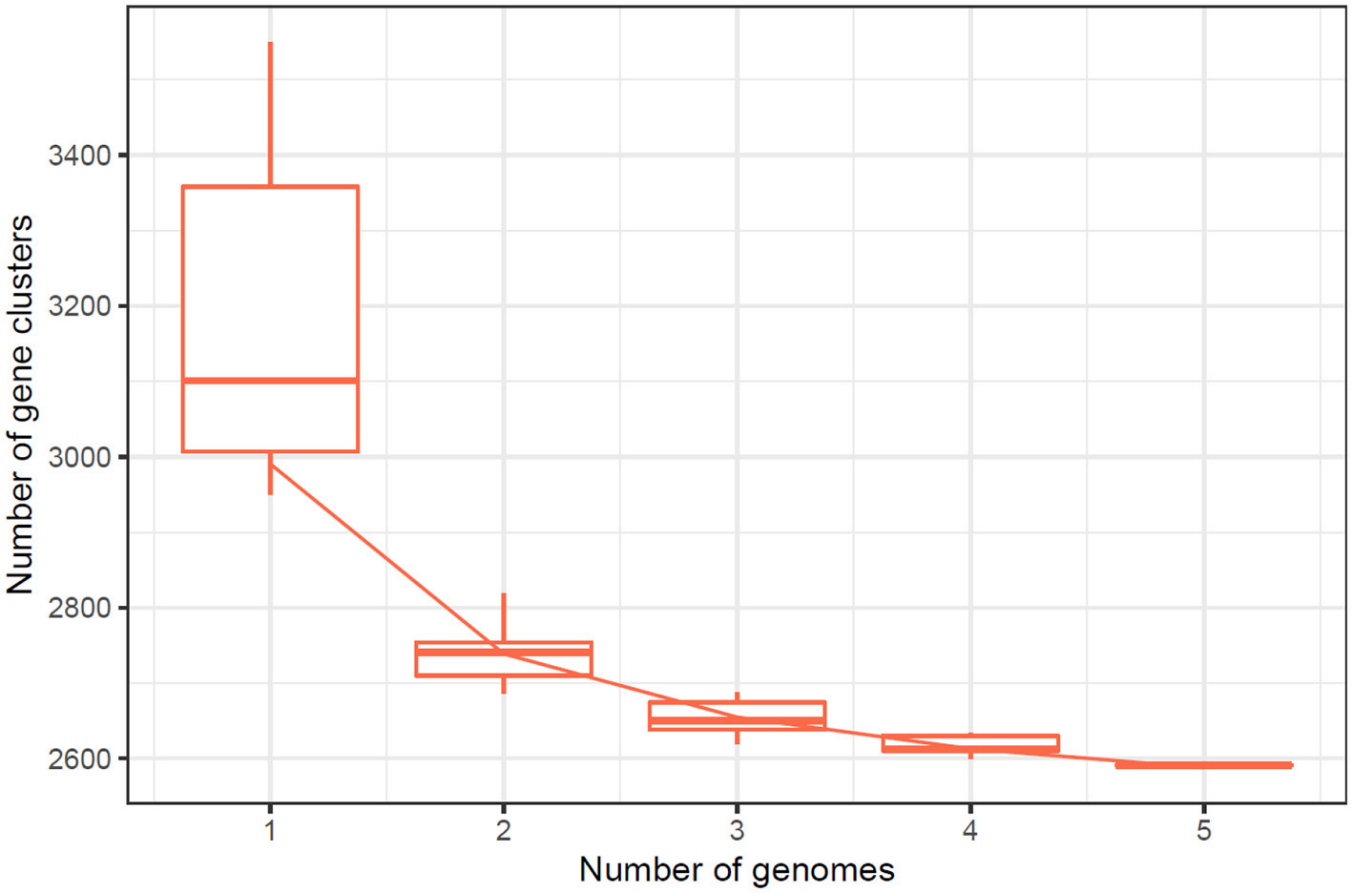
Cluster Cluster Orthologous Groups (COG) results obtained by the pangenome analysis.

**Figure 6:**
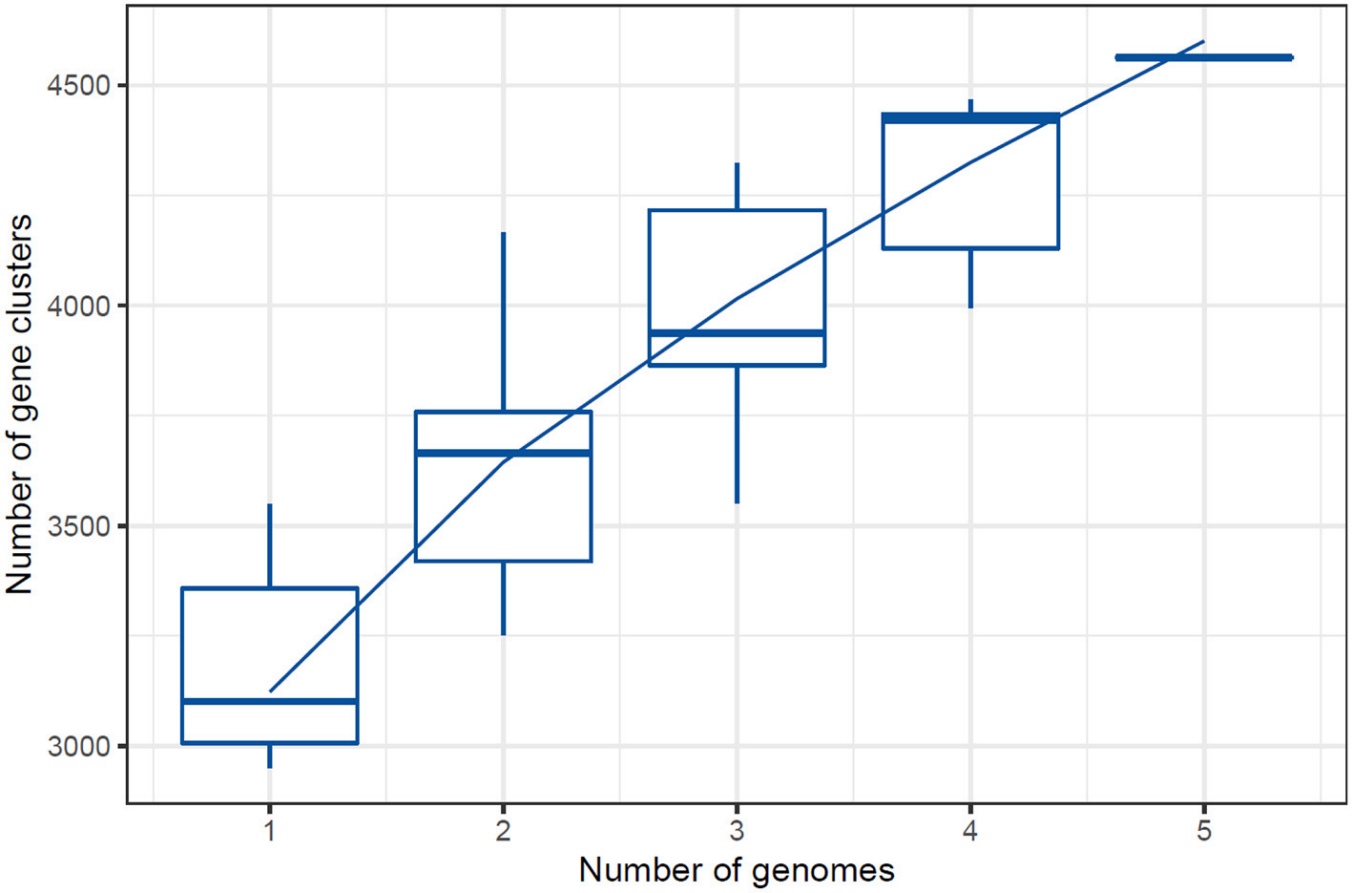
Cluster sharing between five genomes of *Lactiplantibacillus plantarum*.

The quality of the assembled genomes was first analyzed to assess the reliability of the data used in the analysis. Through gene clustering, orthologous groups were defined to clarify the gene content, which differed significantly in functional classification between the core and peripheral pangenome. It was also observed that the cluster share analysis identified core genes present in all genomes, while accessory genes are found only in some genomes. These differences in gene content among the compared genomes were highlighted using tools available in IPGA, revealing the direction of genome divergence and potential adaptive strategies within the species under consideration. The results of the cluster-sharing analysis are presented in Figures 5, 6.

**Figure 7:**
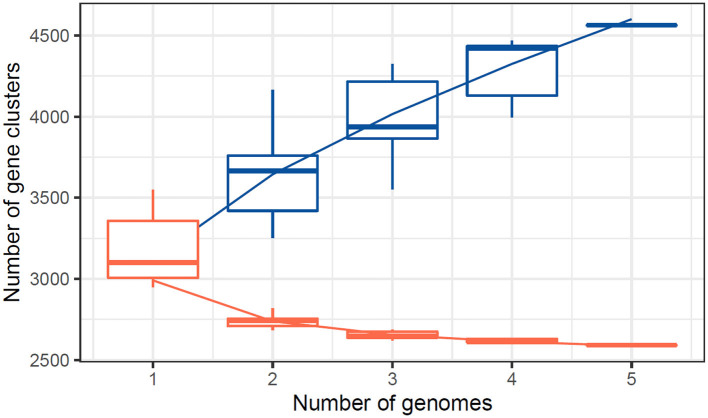
Core pangenome refraction analysis for the studied genomes.

The UpSet plot across the five studied genomes provides an in-depth view of how gene clusters intersect and distribute. The numbers in the left bar plot (*y*-axis) represent each genome's total gene cluster count, ranging from the fewest to the most in the SRCM100442 and DF strains. The top bar plot shows the frequency of intersecting gene clusters, with the largest intersection comprising 2,561 universal core genomes, representing highly conserved genetic content across all genomes. Unique and co-occurring clusters are graphically illustrated in the matrix shown in Figure 7, with black dots indicating specific levels of overlap. For example, 433 gene clusters are exclusively shared between the BDGP2 and DF strains. More localized connections are shown in intersections of smaller sizes, while single dots indicate genome-specific distinct clusters. The plot effectively captures both the relatedness and support for shared conserved gene regions and unique clusters across the genomes (Supplementary Figure S1).

The central pangenome refraction results from the UpSet plot reveal a substantial core genome, with 2,561 gene clusters shared across all five studied genomes. This extensive core genome reflects a high degree of genetic conservation, suggesting that these shared genes are likely crucial for essential cellular functions and survival. Additionally, the plot identifies several unique gene clusters specific to individual genomes and smaller sets of gene clusters shared among subsets of genomes. This highlights genetic diversity and potential adaptive traits unique to certain genomes (Table 6). Combining a strong core genome and diverse accessory genes indicates a balance between conserved essential functions and variable adaptive capabilities across the studied genomes.

**Table 6:** Key genes associated with food safety and probiotic potential.

**Gene**	**Product**	**Function**	**Relation to food safety**	**Relation to probiotic potential**
plnJK, plnEF	Plantaricin JK and EF	Bacteriocin production inhibits pathogen growth	Inhibits foodborne pathogens (*Listeria, Clostridium*)	Provides a competitive advantage in the gut microbiome
MurA	UDP-N-acetylglucosamine 1-carboxyvinyltransferase	Cell wall synthesis maintains bacterial integrity	Enhances survival in food matrices	Enhances survival in the gastrointestinal tract
Alr	Alanine racemase	Peptidoglycan synthesis, maintains cell wall integrity	Enhances bacterial survival in food	Survives in acidic environments such as the stomach
rpoB, rpoC	RNA polymerase beta and beta subunits	Transcription allows adaptation to stress conditions	Supports gene expression under environmental stress	Enhances survival in diverse environments (gut and food)
rho	Transcription termination factor Rho	Gene regulation and stress adaptation	Regulates stress responses, enhancing survival in food	Aids in bacterial adaptation to gut conditions
MprF	L-O-lysylphosphatidylglycerol synthase	Alters cell membrane charge confers resistance to antibiotics	Contributes to bacterial resistance, supporting food safety	Increases resilience in the gut environment
GdpD	Glycerophosphoryl diester phosphodiesterase	Alters cell wall charge confers resistance to environmental stress	Affects membrane stability, enhances pathogen resistance	Contributes to survival and stability in the gut
PgsA	CDP-diacylglycerol-glycerol-3-phosphate 3-phosphatidyltransferase	Alters cell wall charge contributes to antibiotic resistance	Enhances resistance to environmental stress in food environments	Supports resilience in gut conditions and stress resistance

The number of core genes and their distribution patterns change as more genomes are included in the analysis. As shown in the box plots in Figure 7, the number of core gene clusters (in red) decreases sharply and approaches saturation as more genomes are considered. In contrast, the number of pangene clusters (in blue) increases with the discovery of more unique gene clusters as additional genomes are added. These trends illustrate the process of pangenomic diversification and evolution while the core genome content becomes relatively stabilized across the genomes studied. Thus, the analysis contributes to understanding the extent of variation and similarity between species, which is crucial for exploring the evolutionary aspects of their functions. Figure 7 presents the core pangenome refraction analysis results.

### 3.8 Orthologous analysis

The UpSet plot analysis of the *L. plantarum* HMX2 genome indicates substantial intersection patterns between five strains: BDGP2, UNQLp11, DF, HMX2, and SRCM100442. Two of these strains reside outside of chromosome 19. The greatest intersection cluster has 2,559 gene clusters, showing that the tested strains are genetically related. Notably, strain BDGP2 contains the most unique components (3,086), followed by strains DF and HMX2, which have 2,987 and 2,948, respectively. Strains SRCM100442 and UNQLp11 share many elements, revealing their high genetic similarity. Supplementary Figure S2 shows the UpSet plot for the five *L. plantarum* strains.

The cluster Venn diagram study of the *L. plantarum* genome shows the distribution and intersection of protein clusters in five strains: BDGP2 (Band2 domain G pullin 2), DF (diaphanous), HMX2 (hexaminidase 2), SRCM100442, and UNQLp11. The biggest overlap comprises 2,559 functional units shared by all five strains, totaling 12,884 proteins. This shows that a large percentage of the genome is conserved among various *L. plantarum* strains. Furthermore, strain-specific clusters are detected, with BDGP2 having distinct clusters that presumably contribute to its high protein count. All strains contribute nearly equally in terms of protein distribution, while BDGP2, HMX2, and UNQLp11 have a greater number of unique proteins. These findings highlight the genetic variations and the shared and distinct genes across the *L. plantarum* strains, reflecting both the individual characteristics of each strain and the common genes essential for the organism's survival and functionality. Supplementary Figure S3 represents the cluster Venn diagram, showing the distribution and intersection of protein clusters across the five strains.

### 3.9 Prediction of specialty genes

The whole-genome comparison showed genetic variations not found by the 16S rRNA gene approach, resulting in the identification of 25 antibiotic-resistance genes, emphasizing the need for investigating these features for food safety. Among the antibiotic resistance genes discovered are MurA and Alr, which encode the enzymes UDP-*N*-acetylglucosamine 1-carboxyvinyltransferase and alanine racemase, respectively. Both are important antibiotic targets in sensitive bacterial strains. Other important genes include kasA, rpoB, rpoC, gyrB, and gyrA, which encode enzymes essential for bacterial life, such as RNA polymerase and DNA gyrase, resulting in a wide variety of antibiotic resistance.

In addition, other transporter-related genes were found, including putative proteins and transporters, such as glycerol uptake facilitator proteins, which belong to the major intrinsic protein (MIP) family and are involved in nutrition and ion transport. The inclusion of genes such as GdpD and MprF, which modulate cell wall charge to aid antibiotic resistance, adds to *L. plantarum* HMX2's usefulness.

As a result, the whole-genome study of *L. plantarum* HMX2 sheds light on the strain's genetic diversity and possible function in foodborne pathogens and probiotics. These findings open the path for further research into the strain's antibiotic-resistance genes and their functional importance. Supplementary Table S2 shows the main specialized genes found in the *L. plantarum* HMX2 genome.

### 3.10 Food safety and probiotic potential

The genomic investigation of *L. plantarum* revealed numerous important genes involved in food safety and probiotic activity. Notably, the plnJK and plnEF genes, which produce plantaricin, were discovered to have an important role in suppressing foodborne pathogens such as Listeria, adding to the bacterium's antibacterial capabilities in fermented foods. Furthermore, genes, such as MurA and Alr, are required for cell wall formation, improving bacterial integrity, and guaranteeing survival in tough environments seen in food matrices and the gastrointestinal system. Furthermore, transcription-related genes such as rpoB, rpoC, and rho have been found, allowing *L. plantarum* to respond to environmental stress, which is critical for both food preservation and probiotic resilience. The presence of MprF, GdpD, and PgsA genes, which help to change the cell membrane and wall charge, increases resistance to environmental stressors, including antibiotics, allowing the bacteria to survive in food processing conditions and during gut colonization. These findings emphasize *L. plantarum*'s dual role in protecting food safety through antimicrobial activity while increasing probiotic potential by surviving in the gastrointestinal environment.

### 3.11 Protein interaction network

The STRING-based PPI network analysis of the input proteins (glpF6, fusA2, glpF1, rho, orf2, yidC1, and tuf) revealed multiple statistically significant connections. Except for the hypothetical protein ORF2, all proteins are linked together by edges representing particular and important interactions. glpF6 and glpF1, which encode MIP/aquaporin family members, may serve as diffusive transporters for substrates such as lactic acid, urea, and H_2_O_2_.

**Figure 8:**
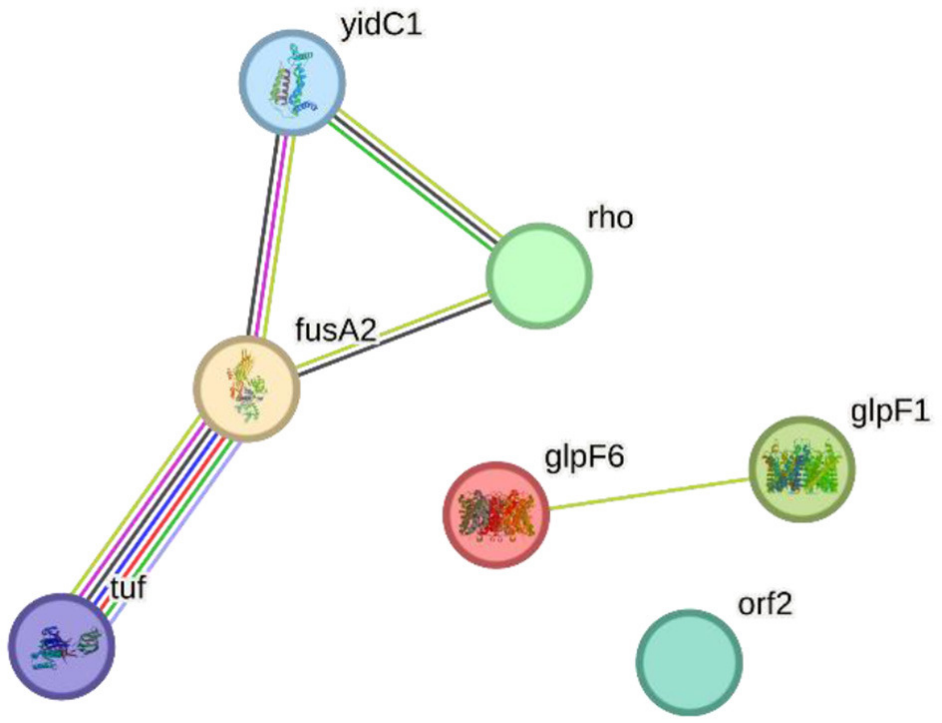
The Search Tool for the Retrieval of Interacting Genes/Proteins (STRING) network indicating the protein interactions of proteins encoded by specialty genes in *Lactiplantibacillus plantarum* HMX2.

Two more genes, fusA2 and tuf, are involved in translation elongation, and their interaction implies a function in protein synthesis. Rho, a transcription terminator factor, co-transcribes with fusA2, suggesting a link between transcription termination and translation elongation. Additionally, yidC1, which is involved in membrane protein insertion, is also linked to fusA2 and tuf, implying their joint involvement in incorporating proteins essential for translation. This study demonstrates how these proteins are functionally interrelated in critical cellular processes such as transcription termination, translation elongation, and membrane protein insertion by dissecting the protein—protein interactome, notably the Hcm1-Sac3 complex. Figure 8 shows the PPI network produced from STRING analysis.

### 3.12 Gene co-occurring analysis

**Figure 9:**
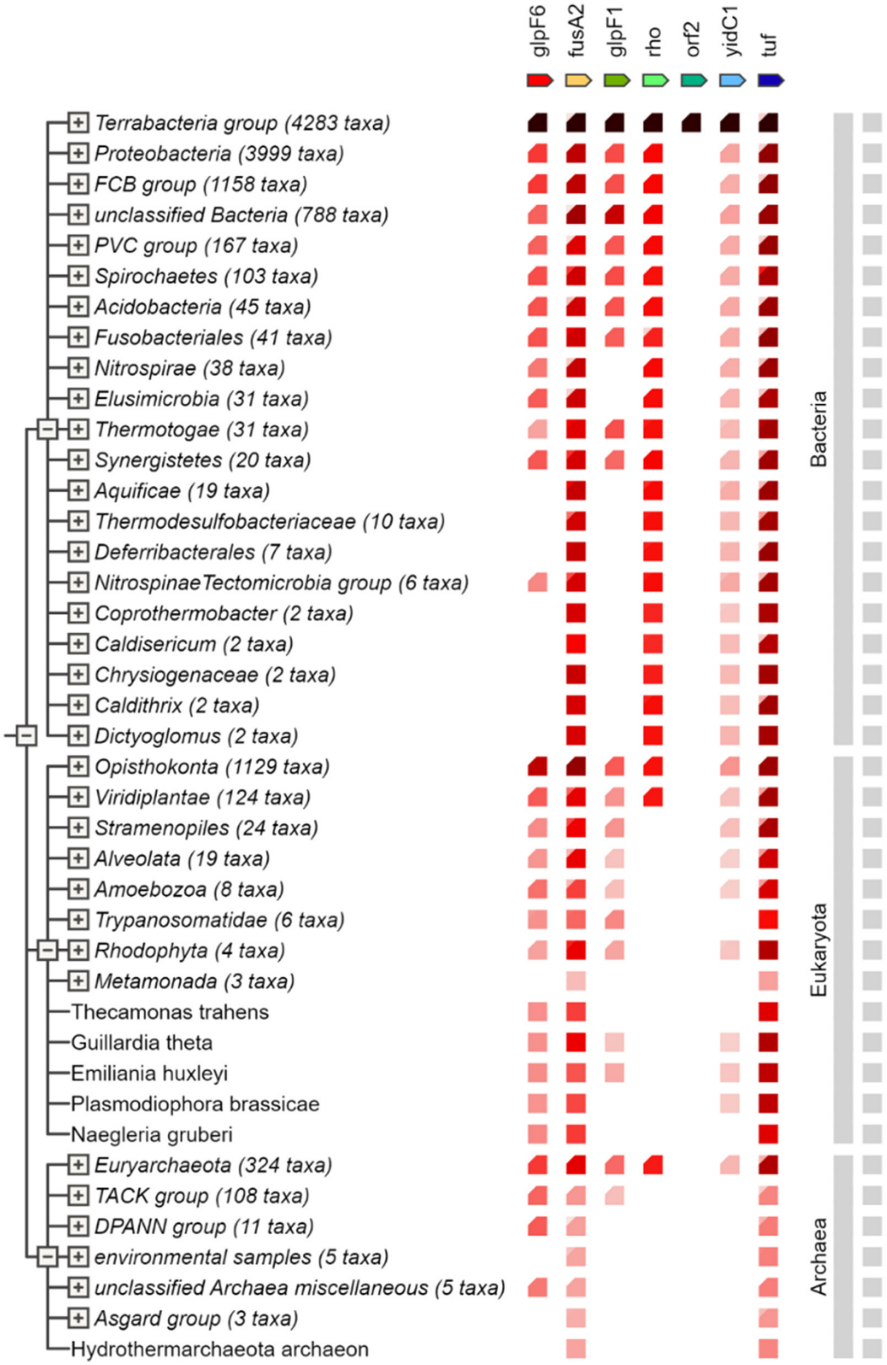
Gene co-occurrence analysis of the specialty genes present in genome of *Lactiplantibacillus plantarum* HMX2.

The major clusters described reflect numerous bigger “functional groups” that arise in various genomes and typically contain genes that co-occur. GlpF6, a putative transporter protein, is closely associated with genes involved in metabolic processes and transport. Similarly, considerable co-occurrence was found for fusA2 and tuf, which are involved in translation elongation. Rho is largely associated with genes that regulate transcription and translation, showing its importance in gene expression. YidC1 interacts with genes involved in membrane protein insertion, indicating that it functions in protein organization and integration inside the membrane. In contrast, the putative protein orf2 has few connections, indicating that it may have a more specialized or context-dependent role. Figure 9 depicts the co-occurrence analysis of the specialty genes present in the genome of *L. plantarum* HMX2.

## 4 Discussion

The genomic features of *L. plantarum* HMX2 have been comprehensively analyzed using the WGS and bioinformatics. These discoveries lead to important information about its genetic, functional, and evolutionary relationships, laying the foundation for the future research into its various uses. These results provide us with a better understanding of the genetics of *L. plantarum* and its implications in food research, probiotics, and antibiotic research. Subsequent studies will focus on using these findings in developing biotechnological advancements and evaluating their work to improve food safety and health (Aziz et al., 2023b). The high genomic similarity (99.17% ANI) between *L. plantarum* HMX2 and the reference strain Z.61 confirmed that it is a member of the *L. plantarum* species. This relationship is consistent with other studies that have found differences in the genetic diversity of *L. plantarum* species in different locations. Similar genetic markers have been found in Lactobacillus bacteria. Additionally, 25 potential antibiotic genes in *L. plantarum* HMX2 deserves a detailed study. Although these genes are present in *L. plantarum*, it is important to understand their expression patterns and their impact on food safety practices. Although this similarity supports its taxonomic status, further research is needed to explore the functional properties of *L. plantarum* HMX 2 (Elagamey et al., 2023).

Other species of *L. plantarum* have been shown to have similar genetic characteristics, including the ability to withstand stress, adapt to the environment, and compete with pathogens. In addition, since there are 25 putative antibiotic resistance genes in *L. plantarum* HMX2, it needs to be checked in detail. This is consistent with previous studies on the diversity of *L. plantarum* and supports the concept of the bacterial pangenome. The evolutionary relationships of *L. plantarum* HMX2 were elucidated using phylogenetic analysis and placed them in a group of related individuals. This underscores the need for research studies to demonstrate the beneficial effects of similar Lactobacillus species (Lu et al., 2024).

Thus, the comparative genomic analysis of the *L. plantarum* strains brought up significant genetic relatedness and differences that revealed much about their evolutionary history and roles (Aziz et al., 2023c). The OrthoANIu value was at a high of 99. Hence, 17% implies that the studied species share a high degree of genome relatedness and testify to high proportions of nucleotide identity within the species. The draft genome and annotation of *L. plantarum* HMX2 strain brought out the genetic plan of the economically beneficial *L. plantarum* highly descriptive with 3, 242 CDS, 65 tRNA, and 16 rRNA genes, many among them were Hypothetical proteins, indicating possibilities for new research in the future. This detailed genomic information helps to develop a better view of the strain's functional potential and its evolutionary history. The analysis for the pangenome revealed a large core genome with 2,561 gene clusters, which could be attributed to the large number of orthologous genes representing the degree of genetic conservation in an organism of basic cellular processes. The finding of specialty genes such as antibiotic resistance, transporters, and other resistance genes provides the light of adaptation to the microbial strain and its significance to food safety and probiotics. These all-encompassing genomic differences and phylogenetic relationships constitute as a strong foundation for the genetic and evolutionary systems within *L. plantarum*, opening up opportunities for future scientific advances in its functional and adaptive characteristics.

This study represents new insights into *L. plantarum* HMX2 genomics, providing knowledge about its genomic features and possible relationship with antibiotic resistance (Hu et al., 2023). It is worthy of note that the identification of the 25 antibiotic resistance genes together with their resistance mechanisms we reveal the ability of the strain to resist several antimicrobial agents which has several important implications for food safety and public health. The phylogenetic and pangenomic analyses not only reveal how various *L. plantarum* strains evolved but also expose the core and accessory genes required for life and versatility. These results highlight the need to further research into microbial genomes to uncover the functionality complexity the different microbial species, especially in the domains of probiotics and foodborne pathogens. This kind of research is important mainly to create effective tactics of how to fight against antibiotic resistance and enhance food security (Contente et al., 2024; Aljohani et al., 2024).

The future prospects of this study are vast and intriguing, with several options for further research. One important area is the experimental confirmation of the antibiotic resistance genes discovered in *L. plantarum* HMX2, which will be critical in assessing the strain's safety and appropriateness for food applications. Comparative genomics may also be used to explore functional diversity across several *L. plantarum* strains, providing further insight into their probiotic potential. The future studies might also examine how the strain interacts with the human gut microbiome to better understand its health advantages and potential uses in functional foods.

## 5 Conclusions

In conclusion, *L. plantarum* has significantly contributed to genome analysis, genetic diversity research, and food safety applications. The discovery of antibiotic resistance genes in *L. plantarum* emphasizes its critical role in protecting food safety. The future comparative genomic investigations are likely to give useful insights into *L. plantarum*'s functional diversity and evolutionary dynamics, increasing its potential as a probiotic and broadening its applicability in developing innovative antimicrobial therapies. This thorough understanding of the *L. plantarum* genome provides a solid framework for its use in various industrial and manufacturing processes, ultimately boosting its incorporation into food production and safety measures.

## Data Availability

The datasets presented in this study can be found in online repositories. The names of the repository/repositories and accession number(s) can be found in the article/Supplementary material.
