# Supplementary material for: Unveiling the whole genomic features and potential probiotic characteristics of novel Lactiplantibacillus plantarum HMX2
Source: Front Microbiol. 2024 Nov 14;15:1504625. doi: 10.3389/fmicb.2024.1504625 (PMC11602494; doi:10.3389/fmicb.2024.1504625)

**Table S1: The presence of Antibiotic Resistance Genes in L. plantarum HMX2 genome**

|  | |
| --- | --- |
| AMR Mechanism | Genes |
| Antibiotic target in susceptible species | Alr, Ddl, EF-G, EF-Tu, folA, Dfr, folP, gyrA, gyrB, inhA, fabI, Iso-tRNA, kasA, MurA, rho, rpoB, rpoC, S10p, S12p |
| Antibiotic target modifying enzyme | RlmA(II) |
| Antibiotic target replacement protein | FabK |
| Gene conferring resistance via absence | gidB |
| Protein altering cell wall charge conferring antibiotic resistance | GdpD, MprF, PgsA |

Table S2: Key specialty genes identified in the genome of L. plantarum HMX2

| Gene | Property | Product | Function | Classification |
| --- | --- | --- | --- | --- |
|  | Transporter | hypothetical protein | the plantaricin jk (plantaricin jk) family. | 1.C.30.1.1 |
| MurA | Antibiotic Resistance | UDP-N-acetylglucosamine 1-carboxyvinyltransferase (EC 2.5.1.7) | UDP-N-acetylglucosamine 1-carboxyvinyltransferase (EC 2.5.1.7) | antibiotic target in susceptible species |
| MurA | Antibiotic Resistance | UDP-N-acetylglucosamine 1-carboxyvinyltransferase (EC 2.5.1.7) | UDP-N-acetylglucosamine 1-carboxyvinyltransferase (EC 2.5.1.7) | antibiotic target in susceptible species |
| Alr | Antibiotic Resistance | Alanine racemase (EC 5.1.1.1) | Alanine racemase (EC 5.1.1.1) | antibiotic target in susceptible species |
| kasA | Antibiotic Resistance | 3-oxoacyl-[acyl-carrier-protein] synthase, KASII (EC 2.3.1.179) | 3-oxoacyl-[acyl-carrier-protein] synthase, KASII (EC 2.3.1.179) | antibiotic target in susceptible species |
| rpoB | Antibiotic Resistance | DNA-directed RNA polymerase beta subunit (EC 2.7.7.6) | DNA-directed RNA polymerase beta subunit (EC 2.7.7.6) | antibiotic target in susceptible species |
|  | Transporter | Glycerol uptake facilitator protein @ Propanediol diffusion facilitator | the major intrinsic protein (mip) family. | 1.A.8.2.5 |
| Iso-tRNA | Antibiotic Resistance | Isoleucyl-tRNA synthetase (EC 6.1.1.5) | Isoleucyl-tRNA synthetase (EC 6.1.1.5) | antibiotic target in susceptible species |
| Ddl | Antibiotic Resistance | D-alanine--D-alanine ligase (EC 6.3.2.4) | D-alanine--D-alanine ligase (EC 6.3.2.4) | antibiotic target in susceptible species |
| rho | Antibiotic Resistance | Transcription termination factor Rho | Transcription termination factor Rho | antibiotic target in susceptible species |
| gyrB | Antibiotic Resistance | DNA gyrase subunit B (EC 5.99.1.3) | DNA gyrase subunit B (EC 5.99.1.3) | antibiotic target in susceptible species |
| S10p | Antibiotic Resistance | SSU ribosomal protein S10p (S20e) | SSU ribosomal protein S10p (S20e) | antibiotic target in susceptible species |
| EF-G | Antibiotic Resistance | Translation elongation factor G | Translation elongation factor G | antibiotic target in susceptible species |
| GdpD | Antibiotic Resistance | Glycerophosphoryl diester phosphodiesterase (EC 3.1.4.46) | Glycerophosphoryl diester phosphodiesterase (EC 3.1.4.46) | protein altering cell wall charge conferring antibiotic resistance |
| RlmA(II) | Antibiotic Resistance | 23S rRNA (guanine(748)-N(1))-methyltransferase (EC 2.1.1.188) | 23S rRNA (guanine(748)-N(1))-methyltransferase (EC 2.1.1.188) | antibiotic target modifying enzyme |
| rpoC | Antibiotic Resistance | DNA-directed RNA polymerase beta' subunit (EC 2.7.7.6) | DNA-directed RNA polymerase beta' subunit (EC 2.7.7.6) | antibiotic target in susceptible species |
| MprF | Antibiotic Resistance | L-O-lysylphosphatidylglycerol synthase (EC 2.3.2.3) | L-O-lysylphosphatidylglycerol synthase (EC 2.3.2.3) | protein altering cell wall charge conferring antibiotic resistance |
| folA, Dfr | Antibiotic Resistance | Dihydrofolate reductase (EC 1.5.1.3) | Dihydrofolate reductase (EC 1.5.1.3) | antibiotic target in susceptible species |
| inhA, fabI | Antibiotic Resistance | Enoyl-[acyl-carrier-protein] reductase [NADH] (EC 1.3.1.9) | Enoyl-[acyl-carrier-protein] reductase [NADH] (EC 1.3.1.9) | antibiotic target in susceptible species |
|  | Transporter | hypothetical protein | the plantaricin ef (plantaricin ef) family. | 1.C.29.1.1 |
|  | Transporter | hypothetical protein | the lactococcin a (lactococcin a) family. | 1.C.22.1.6 |
| gyrA | Antibiotic Resistance | DNA gyrase subunit A (EC 5.99.1.3) | DNA gyrase subunit A (EC 5.99.1.3) | antibiotic target in susceptible species |
| FabK | Antibiotic Resistance | Enoyl-[acyl-carrier-protein] reductase [FMN, NADH] (EC 1.3.1.9), FabK => refractory to triclosan | Enoyl-[acyl-carrier-protein] reductase [FMN, NADH] (EC 1.3.1.9), FabK => refractory to triclosan | antibiotic target replacement protein |
| pox5 | Drug Target | Pyruvate oxidase (EC 1.2.3.3) | Thiamin Diphosphate;Flavin adenine dinucleotide | Drug target |
|  | Transporter | Glycerol uptake facilitator protein | the major intrinsic protein (mip) family. | 1.A.8.2.4 |
| gidB | Antibiotic Resistance | 16S rRNA (guanine(527)-N(7))-methyltransferase (EC 2.1.1.170) | 16S rRNA (guanine(527)-N(7))-methyltransferase (EC 2.1.1.170) | gene conferring resistance via absence |
| folP | Antibiotic Resistance | Dihydropteroate synthase (EC 2.5.1.15) | Dihydropteroate synthase (EC 2.5.1.15) | antibiotic target in susceptible species |
|  | Transporter | PTS system, mannose-specific IIC component | the pts mannose-fructose-sorbose (man) family. | 4.A.6.1.6 |
|  | Transporter | hypothetical protein | the plantaricin jk (plantaricin jk) family. | 1.C.30.1.1 |
| EF-Tu | Antibiotic Resistance | Translation elongation factor Tu | Translation elongation factor Tu | antibiotic target in susceptible species |
|  | Transporter | hypothetical protein | the plantaricin ef (plantaricin ef) family. | 1.C.29.1.1 |
|  | Transporter | Glycerol uptake facilitator protein | the major intrinsic protein (mip) family. | 1.A.8.2.6 |
|  | Transporter | Glycerol uptake facilitator protein | the major intrinsic protein (mip) family. | 1.A.8.2.8 |
|  | Transporter | Integral membrane protein | the yibe/f (yibe/f) family. | 9.B.141.1.2 |
|  | Transporter | Glycerol uptake facilitator protein | the major intrinsic protein (mip) family. | 1.A.8.2.7 |
|  | Transporter | hypothetical protein | the p-type atpase (p-atpase) superfamily. | 3.A.3.3.3 |
| PgsA | Antibiotic Resistance | CDP-diacylglycerol--glycerol-3-phosphate 3-phosphatidyltransferase (EC 2.7.8.5) | CDP-diacylglycerol--glycerol-3-phosphate 3-phosphatidyltransferase (EC 2.7.8.5) | protein altering cell wall charge conferring antibiotic resistance |
| GdpD | Antibiotic Resistance | Glycerophosphoryl diester phosphodiesterase (EC 3.1.4.46) | Glycerophosphoryl diester phosphodiesterase (EC 3.1.4.46) | protein altering cell wall charge conferring antibiotic resistance |
|  | Transporter | Aquaporin Z | the major intrinsic protein (mip) family. | 1.A.8.13.3 |
| S12p | Antibiotic Resistance | SSU ribosomal protein S12p (S23e) | SSU ribosomal protein S12p (S23e) | antibiotic target in susceptible species |

**Figure S1: UpSet plot showing the distribution and intersection of gene clusters among five genomes.**


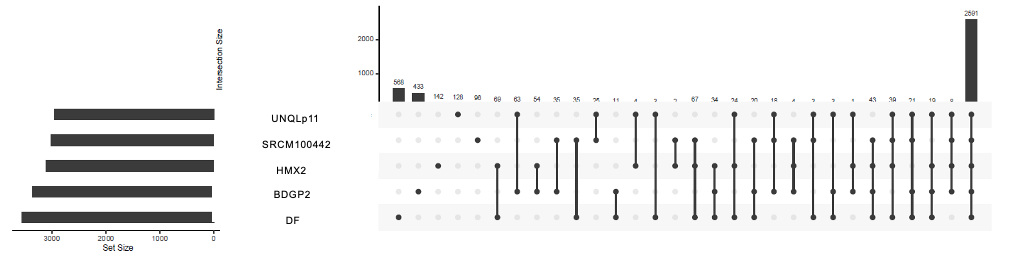


**Figure S2: The Upset Plot for 5 strains L. plantarum species depicting the significant intersection patterns among five strains**


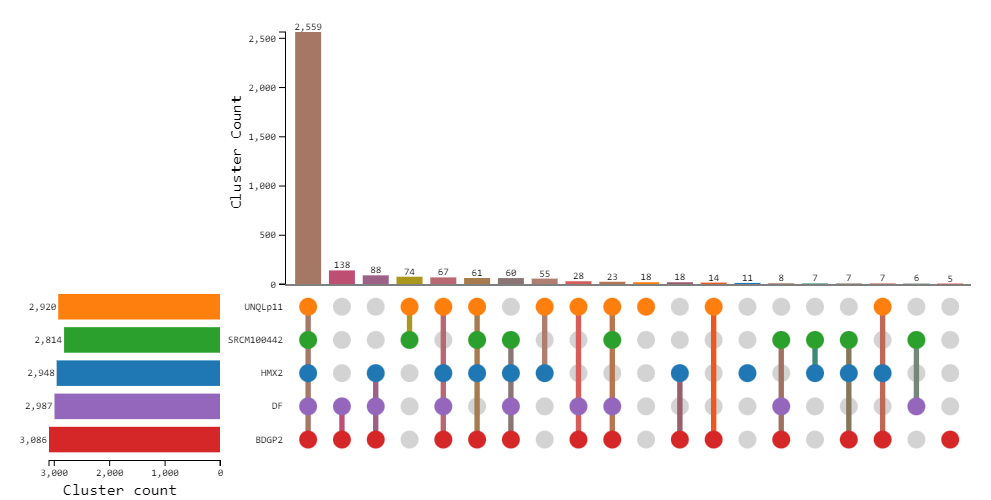


**Figure S3: The cluster Venn diagram of the L. plantarum genome illustrating the distribution and intersection of protein clusters across five strains**


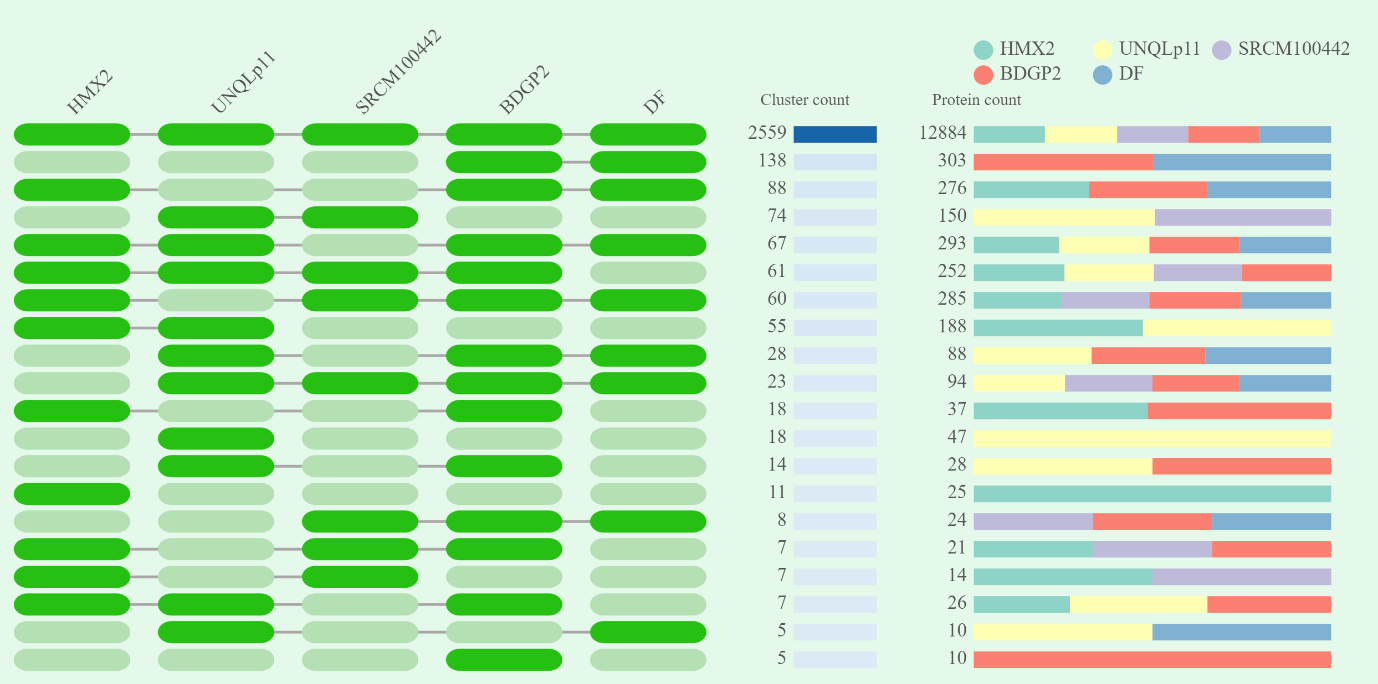

Supplement: Supplementary file 1 [file Data_Sheet_1.docx]
